# Recent Achievements of Self-Healing Graphene/Polymer Composites

**DOI:** 10.3390/polym10020114

**Published:** 2018-01-25

**Authors:** Yongxu Du, Dong Li, Libin Liu, Guangjie Gai

**Affiliations:** School of Chemistry and Pharmaceutical Engineering, Qilu University of Technology (Shandong Academy of Sciences), Jinan 250353, China; Duyongxu2009@163.com (Y.D.); LD18396813772@163.com (D.L.); 18396814715@163.com (G.G.)

**Keywords:** self-healing, graphene, polymer, composite

## Abstract

Self-healing materials have attracted much attention because that they possess the ability to increase the lifetime of materials and reduce the total cost of systems during the process of long-term use; incorporation of functional material enlarges their applications. Graphene, as a promising additive, has received great attention due to its large specific surface area, ultrahigh conductivity, strong antioxidant characteristics, thermal stability, high thermal conductivity, and good mechanical properties. In this brief review, graphene-containing polymer composites with self-healing properties are summarized including their preparations, self-healing conditions, properties, and applications. In addition, future perspectives of graphene/polymer composites are briefly discussed.

## 1. Introduction

Regenerative abilities allow creatures to repair damaged functions to prolong their life span. Researchers are inspired to design and prepare self-healing materials to increase the lifetime of materials and reduce the total cost of systems during the process of long-term use. Recently, great progress has been made in self-healing composite materials that possess the ability to restore their structure and functionality after damage. Early self-healing materials were focused on microcapsule or microtubule by release of healing agents to achieve repairing. However, the self-healing times of these methods are dependent on the amounts of healing agents in the microcapsule or microtubules [1]. To address these limitations, dynamic chemistry involving dynamic covalent chemistry (e.g., imine bonds [2,3], disulfide bonds [4,5,6], acylhydrazone bonds [7,8,9], and boronate ester bonds [7]) and non-covalent interactions, such as hydrogen bonds [10,11], π−π stacking [12], hydrophobic interactions [13,14], host-guest interactions [15], ionic interactions [16], electrostatic interactions [17], and metal-coordination interactions [18,19,20], has been recently introduced to construct self-healing materials with multiple reversible healing ability.

**Figure 1:**
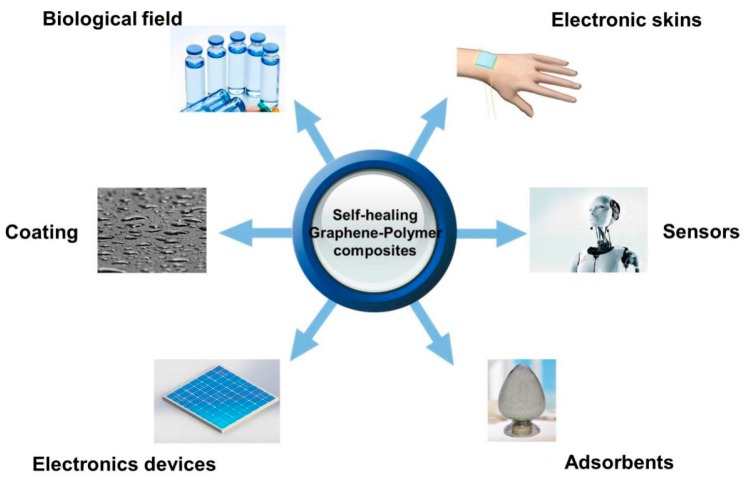
Applications of self-healing graphene/polymer composites.

Graphene, as a new type of two-dimensional planar monolayer of sp^2^ carbon atoms, has attracted widespread attention in all kinds of research areas due to its large specific surface area, excellent electrical conductivity, thermal conductivity, and unique mechanical properties [21,22,23,24,25]. Recently, graphene or graphene derivatives have been widely introduced into polymer matrices. The excellent performance of graphene or graphene derivatives, combined with the advantages of the polymer matrix, makes graphene/polymer composites suitable for application in conductive devices, coating, and biological and pharmaceutical field [26,27,28,29,30]. Although graphene-based composites have been well established [31,32,33,34], graphene-containing composites with self-healing capacity have not been summarized up to now. Introduction of self-healing capability into graphene/polymer composites will endow them with the ability of repairing themselves after damage and enlarge their service life. A lot of studies have been reported on the self-healing of the graphene/polymer composites due to their wide applications (Figure 1). Therefore, it is necessary to review self-healing graphene/polymer composites, which combine the outstanding properties of graphene with advantages of the polymeric matrix and can be used in the field of mechanics, thermology, photology and electricity.

In this review, the current advances in self-healing graphene/polymer composites have been summarized, including their preparation methods, self-healing conditions, properties and applications. Finally, the future prospects of the self-healing graphene/polymer composites are discussed.

## 2. Fabrication Methods

### 2.1. Simple Mixing

The simple mixing is the simplest and commonest method for preparing graphene or graphene derivative/polymer composites [35,36,37,38,39,40,41,42,43]. Usually, graphene or graphene derivatives are blended with polymers by mechanical mixing or ultrasonic dispersion. For example, Sabzi et al. [44] prepared poly(vinyl alcohol) (PVA)/Agar/graphene self-healing hydrogels by simply mechanical stirring and ultrasonication.

**Figure 2:**
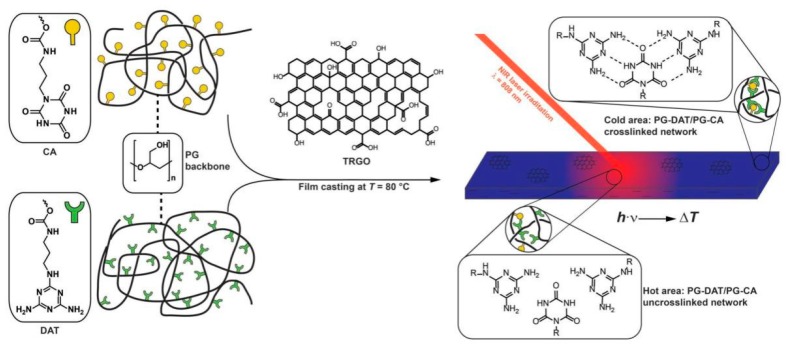
Remote, spatiotemporal, light-fueled modulation of the mechanical properties of co-assembled PG-DAT/PG-CA films hybridized with TRGO. The incorporation of TRGO in low amounts (0.1 wt %) allows localized heating via a NIR laser (808 nm) to break the hydrogen bonds, thus allowing molecular motion and relaxation. Reproduced with permission from [37]. Copyright (2017) Advanced Functional Materials.

Considering its easy agglomerate due to the strong π-π interactions, usually, graphene has been oxidized into graphene oxide (GO). Yan et al. fabricated chitosan/GO supramolecular hydrogels with self-healing properties [45]. It was found that at high GO concentration, a hydrogel can be obtained by simple mixing chitosan and GO at room temperature. However, at low GO concentration, the supramolecular hydrogel formed only at high temperature (95 °C). Walther et al. fabricated rapid self-healing supramolecular elastomers by simple mixing graphene and supramolecular pseudo-copolymer [37]. The copolymer system was formed by co-assembly of diaminotriazine (DAT) functionalized polyglycidols (PG) and cyanuric acid (CA) functionalized PG. Thermally reduced graphene oxide (TRGO) was added from a freshly sonicated dispersion to a heated supramolecular pseudo-copolymer to reach 0.1 wt % in the final nanocomposite. The excellent photothermal effect was enhanced by TRGO, which made the hydrogen bonds break and bond in co-assembled elastomers (Figure 2).

### 2.2. In Situ Polymerization

In situ polymerization can be interpreted to mean that the monomer and graphene or graphene derivative were mixed firstly and subsequently polymerized by the addition of initiators [46,47,48,49,50,51,52,53,54,55,56,57,58]. For example, Green et al. demonstrated physically cross-linked graphene-polyacrylamide (PAM) self-healing hydrogels with increased thermal stability and electrical conductivity [54]. All the reactants, acrylamide (AM), *N*,*N*-methylenebisacrylamide (MBA), and potassium persulfate, were added to the graphene dispersion in water and polymerized.

**Figure 3:**
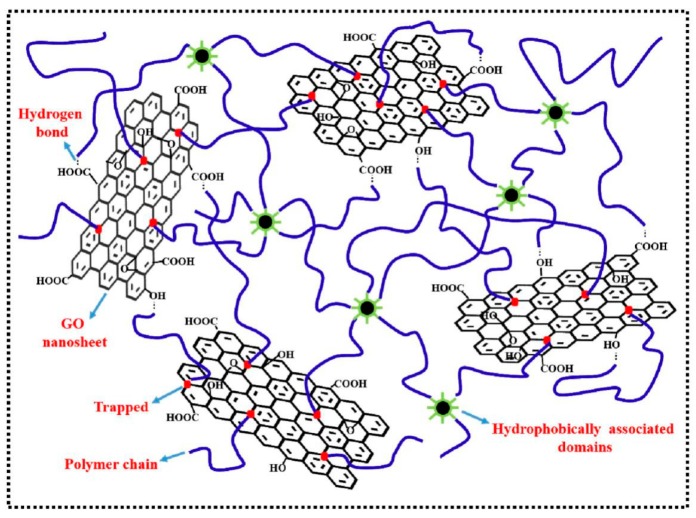
A proposed structure illustration of GO/hydrophobically associated polyacrylamide (HAPAM) composite hydrogels. Reproduced with permission from [49]. Copyright (2015) Journal of Materials Chemistry A.

Taking advantage of this method, our group fabricated cationic PAM/GO self-healing hydrogels with tough, stretchable, compressive property [46]. The GO aqueous dispersion was adjusted to pH 10 by dropping ammonium hydroxide. Successively, the monomers 2-(dimethylamino)ethylacrylatemethochloride (DAC) and AM were added into the GO suspension under stirring followed by the addition of MBA. After adding initiators, polymerization was carried out in an oven at 35 °C for 12 h. Ran et al. fabricated self-healing GO/hydrophobically associated polyacrylamide (HAPAM) composite hydrogels [49]. During the synthesis, GO, the hydrophilic monomer AM and the hydrophobic monomer stearyl methacrylate were mixed to make a uniform solution. After that, potassium persulfate was added to the solution to initiate the polymerization. As shown in Figure 3, a dual cross-linked network was formed after introducing GO into HAPAM through a facile one-pot in situ polymerization.

**Figure 4:**
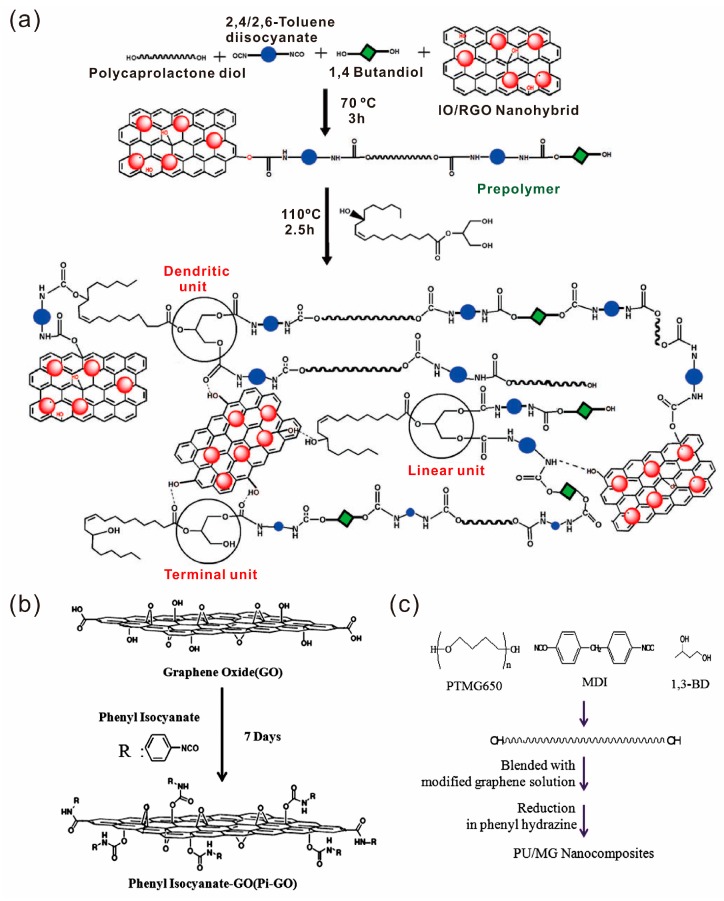
(**a**) Synthesis of a hyperbranched PU/IORGO nanocomposite. Reproduced with permission from [59]. Copyright (2015) New Journal of Chemistry. (**b**) Modification of graphene oxide (GO) by phenyl isocyanate. (**c**) Overall reaction scheme to prepare a PU/MG nanocomposite. Reproduced with permission from [62]. Copyright (2013) European Polymer Journal.

During the in situ polymerization process, graphene can also be modified and participate in the polymerization, forming the covalent bond between graphene and polymers. For example, Karak et al. fabricated a tough self-healing elastomeric nanocomposite containing a castor oil-based hyperbranched polyurethane (PU) and an iron oxide nanoparticle decorated reduced graphene oxide (IORGO) nanohybrid [59]. The IORGO was prepared by the co-precipitation of ferrous and ferric ions on the GO sheets, followed by the reduction of GO. The reaction was carried out by in situ polymerization of poly(ε-caprolactone) diol, 1,4-butanediol, 2,4/2,6-toluene diisocyanate and IORGO. After formation of pre-polymer, a monoglyceride of castor oil as a chain extender was added to form the resulting PU/IORGO nanocomposites. The reaction process is a conventional condensation reaction and is shown in Figure 4a. The same group also prepared sulfur nanoparticle decorated reduced graphene oxide (SRGO) [60] and fabricated self-healing hyperbranched PU/SRGO nanocomposites [61].

Similarly, Kim et al. synthesized a phenyl isocyanate modified GO and obtained the self-healing composites by condensation reaction of poly(tetramethylene glycol) and 4,4′-methylene diphenyl diisocyanate and phenyl isocyanate modified GO in the presence of phenylhydrazine [62] (Figure 4b,c).

### 2.3. Diels-Alder (DA) Reactions

**Figure 5:**
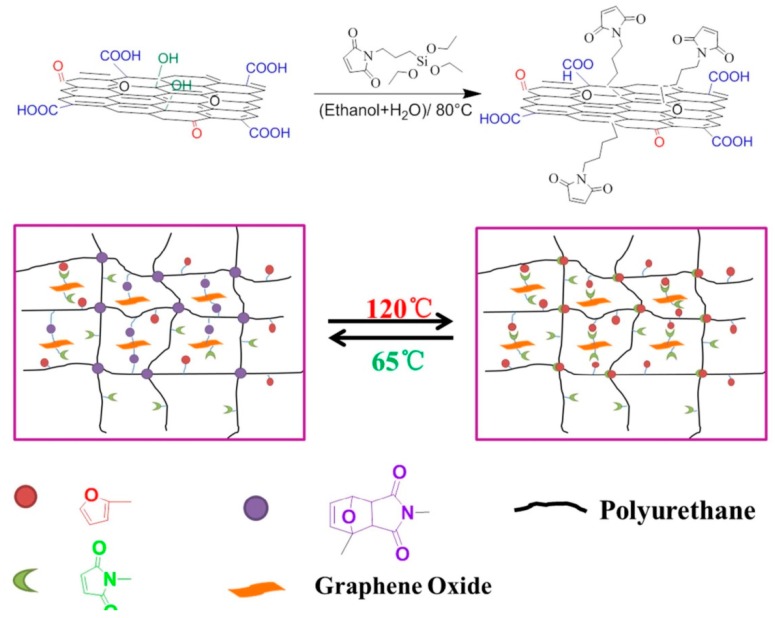
Synthetic routes of nanocomposites. Reproduced with permission from [67]. Copyright (2017) Polymer.

The DA reaction and its retro-Diels-Alder (rDA) analogue is a promising route to introduce self-healing properties to polymeric systems, which can be performed under mild conditions without any catalyst or healing agent [63,64,65,66]. To realize the DA reaction, GO is usually functionalized to react with polymers. For example, Liu and coworkers synthesized the maleimide functionalized GO [67], which can produce a DA cross-linked bond with furan groups of the polyurethane chains (Figure 5). Zhang et al. synthesized an ultrafast self-healing composite material based on DA reactions. The surface modification of graphene involved hydramine-functionalized graphene oxide (FGO) and reduction of FGO to afford hydramine-functionalized graphene nanosheets. The resulted composite was formed by introduction of functionalized graphene nanosheets into the pre-PU which was prepared from the condensation of NCO-terminated PU and a DA resultant of furfury alcohol and bismaleimide [68,69].

### 2.4. Layer-by-Layer Self-Assembly Technique

**Figure 6:**
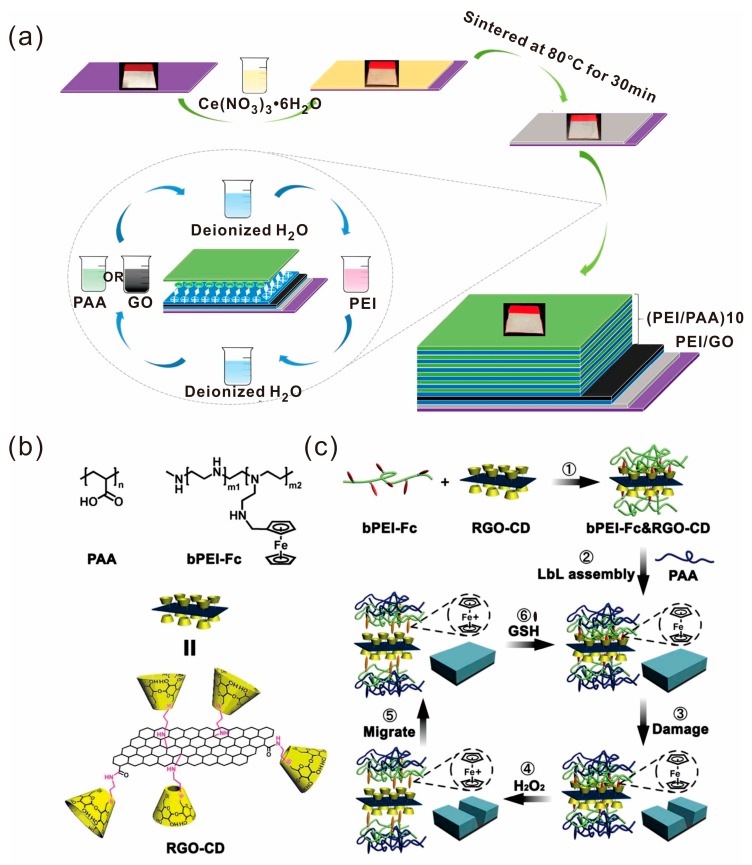
(**a**) Preparation process of the self-healing anticorrosion coating. Reproduced with permission from [75]. Copyright (2015) ACS Applied Materials and Interfaces. (**b**) The chemical structures of bPEI-Fc, PAA and RGO-CD. (**c**) Schematic illustration of the fabrication and healing process of the PAA/bPEI-Fc&RGO-CD composite films. Reproduced with permission from [77]. Copyright (2017) ACS Nano.

The layer-by-layer (LBL) self-assembly technique is a versatile approach to fabricate multilayered nanostructural composites [70,71,72,73]. The first implementation of this technique is attributed to J. J. Kirkland and R. K. Iler, who carried it out using microparticles in 1966 [74]. The LBL self-assembly technique now can be accomplished by using various methods such as immersion, spin, spray, electromagnetism, or fluidics. For example, Fan et al. introduced a self-healing anticorrosion coating on a magnesium alloy (AZ31) substrate [75]. Firstly, cerium nitrate hexahydrate was coated on AZ31 substrate and then the sample was heated at 80 °C for 30 min to partially convert the oxide Ce(III) to Ce(IV). Subsequently, poly(ethyleneimine) (PEI) and GO was coated on the sample to form the PEI/GO layer. Finally, the PEI/GO coated sample was immersed in PEI, deionized water, poly(acrylic acid) (PAA), and deionized water, alternatively (Figure 6a). Graphene oxide was incorporated as corrosion inhibitor and the self-healing ability was attributed to the PEI/PAA multilayers. Ge et al. also prepared a self-healing multilayer polyelectrolyte film based on branched PEI, PAA and graphene by a LBL self-assembly technique [76].

Sun and coworkers reported an intrinsically healable, reduced graphene oxide (RGO)-reinforced polymer composite film via LBL assembly [77]. RGO modified with β-cyclodextrin (β-CD) (denoted as RGO-CD) can complex with branched PEI grafted with ferrocene groups (Fc) (denoted as bPEI-Fc) based on host-guest interactions to form bPEI-Fc&RGO-CD complexes. The bPEI-Fc&RGO-CD complexes are LBL assembled with PAA to fabricate PAA/bPEI-Fc&RGO-CD composite films. The reversible host-guest interactions between nanofillers and LBL-assembled polyelectrolyte films make the composites possess excellent mechanical robustness and highly efficient self-healing properties simultaneously (Figure 6b,c).

### 2.5. Hydrothermal Methods

Apart from the aforementioned fabrication methods, there are other ways to fabricate self-healing graphene/polymer composites. Tang et al. reported conductive and self-healing nanocomposite hydrogels though a simple hydrothermal method [78]. All the reagents were poured into a Teflon-lined stainless steel autoclave and heated at 100 °C for 10 h, forming nanocomposite hydrogels.

## 3. Self-Healing Condition

To restore their original properties, graphene/polymer composites need to repair themselves autonomously or require external energy/stimuli such as mechanical force, light, heat, pH changes. In this section, different self-healing conditions of graphene/polymer composites were summarized.

### 3.1. Heating 

Heating is a common self-healing condition due to its easy operation. By heating, the mobility of polymer chains increases, thus facilitating the self-healing of graphene/polymer composites [40,41,43,48,51,53,54,56,67,79,80,81,82]. Pugno and Valentini’s group fabricated a negative temperature coefficient (exhibiting electrical resistance decrease with temperature increase) silicone rubber (SR)-graphene nanoplatelets (GNPs) composite that can be healed by simple thermal annealing in an oven up to 250 °C for 2 h [43]. After heating treatment, the composite showed a healing efficiency of ~87% by tensile strength. The reversible crosslinking among the damaged network of SR/GNP composite can be thermally activated due to free silanol groups. In conductive composites, the electrical conductivity commonly decreased due to the destroyed conductive network during tensile cycles. However, Zhan et al. reported a conductive graphene/natural rubber composite [40], in which the electrical conductivity rose to nearly two times higher than that of the original one after four tensile cycles and subsequently thermal treatment. The increased conductivity indicates that the destroyed networks, which occur during the tensile process, can be healed during the post thermal treatment.

### 3.2. Light Radiation

**Figure 7:**
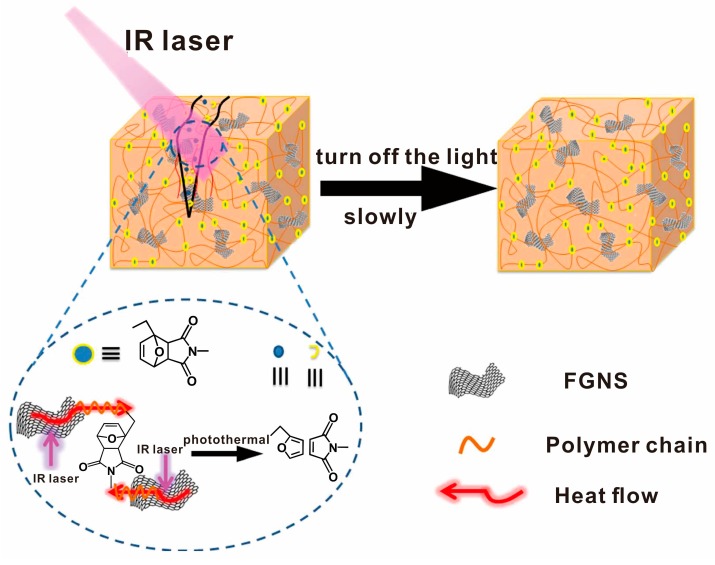
Illustration of the self-healing process of FGNS-PU-DA nanocomposite. Reproduced with permission from [68]. Copyright (2017) ACS Applied Materials and Interfaces.

Although heating is an easy way to repair damaged graphene/polymer composites, during heating other parts of devices are susceptive to heat, thus heating will cause interference of other parts of the devices and result in the deterioration of devices. Therefore, light radiation is an alternative way to repair the damaged composites. Graphene has good ability of photothermal energy transformation that makes the self-healing graphene/polymer composites usually show the same capacity [83]. Zhang et al. synthesized an ultrafast infrared (IR) laser-triggered self-healing composite material [68]. Due to the IR absorbing capacity of functionalized graphene nanosheets (FGNS), the temperature of the composites increased from 30 °C to 150 °C over within 20 s under IR laser irradiation, which reaches the healing temperature of rDA chemistry. The healing efficiency of the snipped specimen reached more than 96% in terms of Young’s modulus, fracture strength, fracture elongation only by IR laser irradiation in 1 min (Figure 7).

The near-infrared (NIR) irradiation has been suggested as a non-invasive, harmless and highly efficient skin-penetrating biomedical technique [84,85]. Tong and coworkers demonstrated a fast self-healing GO-hectorite clay-poly(*N*,*N*-dimethylacrylamide) hybrid hydrogel realized by NIR irradiation for only 2~3 min up to the strength recovery of ~96% [52]. GO acted not only as a collaborative cross-linking agent, but also as a NIR irradiation energy absorber to transform it to thermal energy rapidly and efficiently to promote the mutual diffusion of the polymer chains across the cut interface. Liang and coworkers reported a self-healing bilayer hydrogel system [86]. When the fracture surfaces of the cut hydrogel were contacted, the healing is achieved by irradiating with a NIR illuminant with a wavelength of 808 nm and a power of 1.25 W. The self-healing behavior was ascribed to the photothermal energy transformation property of GO. With the increase of GO content, the heating rate of the hydrogels increased. Kim et al. synthesized self-healing PU/graphene nanocomposites with mechanical, thermal, optical properties [62]. The self-healing ability of PU/graphene nanocomposites was achieved because of intermolecular diffusion of polymer chains, which can be accelerated by NIR absorptions.

Considering NIR as a non-invasive, harmless and highly efficient biomedical technique, it can be used in photothermal therapy. Wang et al. reported a hydrogel made by cross-linking poly(*N*,*N*-dimethylacrylamide) chains on 3D graphene networks that exhibits good neural compatibility, high conductivity, low impedance and efficient NIR-triggered photothermal self-healing [57,87]. The self-healing hydrogel can act effectively as a promising artificial tissue material. 

In addition to IR or NIR, other wavelengths of light radiation can also achieve self-healing capability. For example, Fei et al. fabricated a tri-layered, light-triggered healable and highly electrically conductive fibrous membrane by depositing RGO and silver nanowires onto gold nanoparticles incorporated poly(ε-caprolactone). The polymer chains interdiffuse across the crack surface of the damaged fibrous membrane under 532 nm light irradiation, and recrystallize upon cooling after turning off the continuous-wave diode laser. The surface conductivity recovery by 91% and tensile strength of the membrane are still well maintained after multiple cutting-healing cycles [88].

### 3.3. Microwave

Graphene has an excellent microwaves absorption capability due to its large area conjugated π-structure [89,90]. Under microwaves absorption, the π-structure of graphene will make it a giant electric dipole and transform microwaves into heat in the form of dipoles distortion [91], thus the microwaves absorption can also be used for self-healing of the graphene/polymer composites. For example, Zhang et al. utilized DA chemistry to prepare covalently crosslinked reduced functionalized GO/PU composites with self-healing ability using microwaves [63]. The two broken surfaces were immediately reunited when subjected to a gentle pressure and exposed to a 800 W domestic microwave oven operating at 2.45 GHz for 5 min followed by 2 h at 70 °C without any continuous pressure. The microwaves absorbed by reduced functionalized GO turned into heat and then promoted the healing process of the composites based on DA chemistry. The healed samples still possess good Young’s modulus, fracture strain and fracture stress. Iron oxide (IO) nanoparticles have intrinsic microwave absorbing capacity and high thermal conductivity, which can also be incorporated in self-healing composites. Karak et al. reported a tough self-healing elastomeric nanocomposite containing IORGO, which exhibits the healing efficiency of 99% or more by microwave treatment [59].

### 3.4. Solvent-Assistant Self-Healing

**Figure 8:**
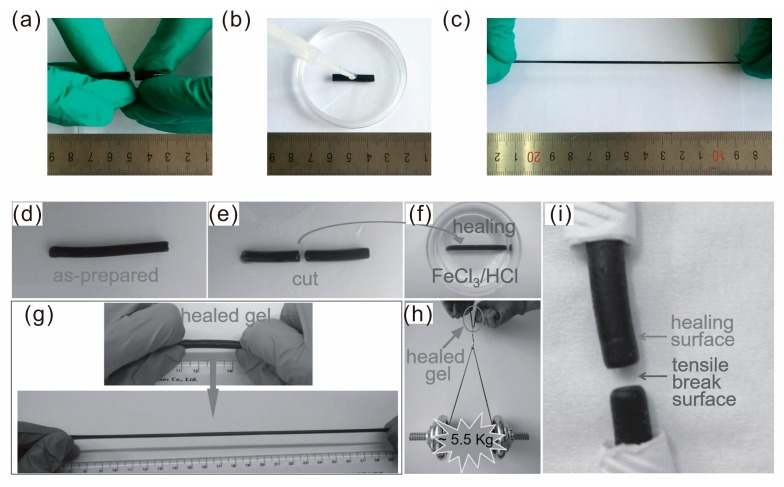
Self-healing properties of hydrogels. A pristine cylinder of the sample was cut in half (**a**). The two-halves were simply brought into contact, and a drop of water was dropped on the cut surface (**b**). After standing for 24 h, the sample can be stretched to a large strain by hand (**c**). Reproduced with permission from [46]. Copyright (2017) ACS Applied Materials and Interfaces. Photos of the self-healing hydrogel: (**d**) as-prepared; (**e**) cut sample; (**f**) contacted and immersed in FeCl_3_/HCl; (**g**) stretched to ≈600% after immersing in FeCl_3_/HCl for 5 h; (**h**) loaded with ≈5.5 kg after healing for 15 h; (**i**) the healing surface and broken surface after tensile test of the hydrogel healed for 15 h. Reproduced with permission from [47]. Copyright (2016) Macromolecular Materials and Engineering.

Deionized water or organic solvent is a practicable external stimulus to heal damage [71,92,93,94]. Solvents can be helpful to the recombination of chemical bonds. In our group the hydrogels fabricated by copolymerization of AM and DAC in the presence of GO can be healed by dropping of water [46]. The cut pieces were slightly put together with the fracture surfaces contacting each other. Because the fresh fracture surfaces are relatively adhesive when the hydrogel was cut, no additional external force is required for connecting the broken parts. After a drop of water was dropped on the fracture surfaces, the healed sample can be stretched to a large strain by hand (Figure 8a–c). Without water assistance, the healed hydrogel has a fracture stress of 248.9 kPa and the healing efficiency is about 45.6%. After self-healing with water assistance, the hydrogel reaches a stress of 503.4 kPa and the healing efficiency on the base of fracture stress is 92.3%. These results indicate that ionic bonds and hydrogen bonds can be reformed via water assistance.

In addition, salt solution can also facilitate the self-healing process. For example, Wang and Tong et al. prepared a multiple shape memory, self-healable, and super tough PAA-GO-Fe^3+^ hydrogel [47]. The self-healing process is due to the strong ionic binding of Fe^3+^ ions to the carboxyl groups on the PAA chains. Keeping the cut surfaces in contact and immersing them in FeCl_3_/HCl solution for a certain time gave rise to the self-healing process. After 15 h immersion, the healed hydrogel can bear a dumbbell of 5.5 kg. Importantly, the break position of the healed hydrogel after tensile test is not the healing position, proving the perfect connection of cut surfaces by Fe^3+^ ions (Figure 8d–i).

### 3.5. Simple Contacting without Any External Stimuli

This self-healing process is realized at room temperature without any external stimuli, which is more practical considering the economic and operation aspects [78,95,96,97,98,99]. For instance, Peng and Turng et al. fabricated a mussel-inspired electroactive chitosan/GO composite hydrogel [95]. The self-healing property was due to dynamic covalent bonds, hydrogen bonding and π-π stacking. The two pieces of the hydrogel can be reconnected when the fractured surface contacts. The stress–strain curves of the recovered hydrogel almost coincided with that of the original one. Polyborosiloxane (PBS) is a well-known “solid–liquid” material whose viscoelastic properties (it flows as a highly viscous liquid at low strain rates but behaves as a solid at high strain rates) promote fast and complete healing due to its dynamic dative bonds. However Saiz et al. developed self-healing graphene-based PBS composites [98]. The mechanical and electrical properties of the composites can be autonomously and fully healed no matter scratched or complete ruptured just by placing the fracture surfaces in contact at ambient conditions for 10 min without external stimulus. Since the healing is driven by polymer flow and the dynamic dative bond interactions between polymer chains, the self-healing can be repeated multiple times.

**Figure 9:**
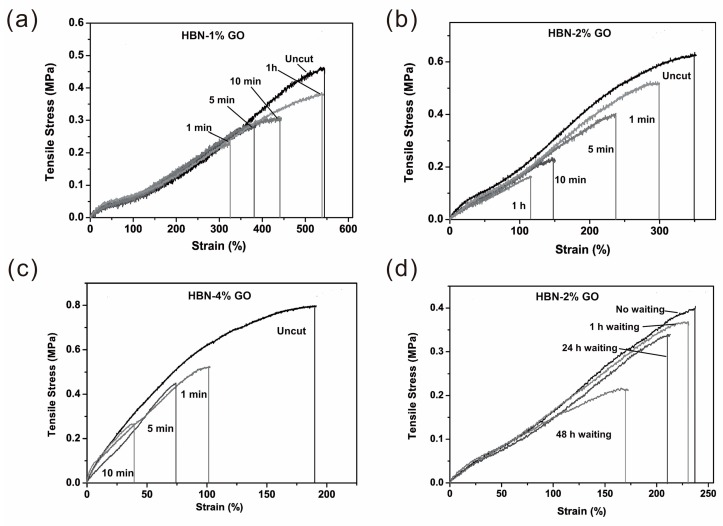
Strain-stress curves of the self-healing composites with GO at (**a**) 1 wt %, (**b**) 2 wt % and (**c**) 4 wt % (termed HBN-1% GO, HBN-2% GO, HBN-4% GO, respectively) upon different healing time at room temperature; (**d**) Strain-stress curves of the HBN-2% GO samples of 10 min healing at different waiting time. The waiting was performed at approx. 0% relative humidity from [99]. Copyright (2013) Advanced Materials.

Self-healing speed is another key factor for practical application. Designing materials with high self-healing speed without any external stimuli is highly desired. Bao et al. synthesized a rapid and efficient self-healing thermo-reversible elastomer (HBN-GO) based on GO and amine-terminated randomly branched oligomers [99]. Both the amorphous structure of the elastomer and its low glass transition temperature (*T*_g_) allow the polymer chains to diffuse and mix at room temperature, and be able to self-heal at room temperature without the need of any plasticizer, solvent, healing agents or external stimuli. Two cut pieces of samples were gently brought into contact and healed in less than 60 s. The obtained stress–strain curves were similar to that of the original uncut samples (Figure 9). The HBN-1% GO sample can be healed to 60% of its original tensile strength prior to cutting by bringing the two cut pieces back in contact for 1 min. In addition, complete healing of the mechanical properties can be obtained in 1 h. Increasing the amount of GO allows for more covalent cross-linking and more restricted movement of polymer chains, which may explain the observed trend for increased time for the self-healing process. Both the samples in HBN-2% GO and HBN-4% GO also possess fast self-healing capabilities, in which they are able to recover 36% and 20% of their original extensibilities, respectively, in 1 min. the self-healing capability only dropped to 90% efficiency after the two fractured surfaces were left apart for 24 h, and dropped further to 50% after 96 h (Figure 9).

Kim et al. developed a mussel-mimetic nanocomposite hydrogel based on catechol-containing polyaspartamide and GO [97]. Two pieces of damaged gels can be strongly healed by contacting with each other due to the dynamic complexation between B^3+^ and catechol. No obvious cut line was seen and the healed sample can be stretched without fracturing by the tweezers.

### 3.6. Microcapsules and Others Healing Methods

**Figure 10:**
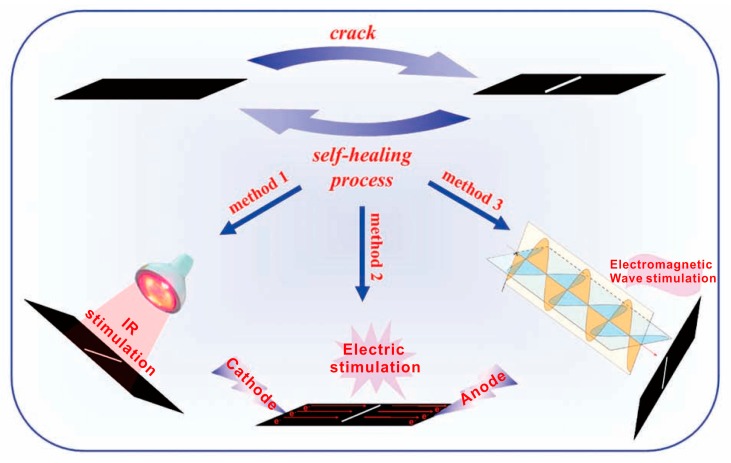
The graphene-thermoplastic PU composites were healed by IR light, electricity and electromagnetic waves with high healing efficiencies. Reproduced with permission from [91]. Copyright (2013) Advanced Materials.

Apart from the aforementioned self-healing conditions, microcapsules are also utilized to heal the damaged composites, although this method cannot realize multiple healing in one damaged area. Gao et al. fabricated GO microcapsules (GOMCs) in Pickering emulsions containing linseed oil as the healing agent [36]. The GO microcapsules were embedded into a waterborne PU matrix to prepare self-healing and anticorrosive coatings. The self-healing of graphene/polymer composites can be achieved through multiple methods. For example, Huang and Chen et al. synthesized mechanical enhanced graphene-thermoplastic PU composites [91], which can be self-healed by IR light, electricity and electromagnetic waves with high healing efficiencies (Figure 10). Zhang et al. also reported a recyclable epoxy resin (ER)/graphene nanocomposite, where a graphene crosslinked ER matrix via DA reaction can be rapidly and efficiently healed via multiple approaches, including heat, IR light, and microwave [35]. 

## 4. Properties and Applications

### 4.1. Mechanical Property

**Figure 11:**
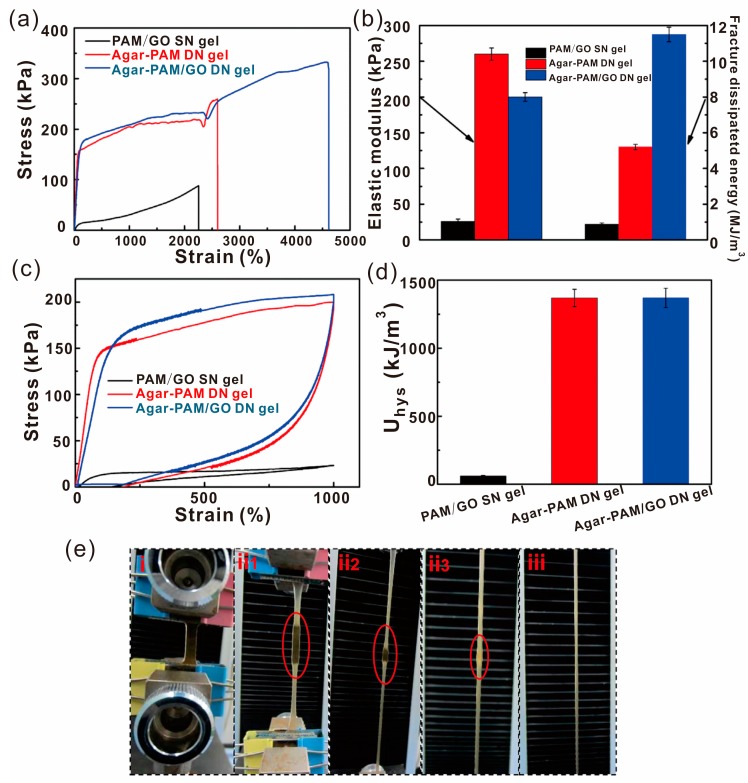
(**a**) Tensile stress–strain curves, and (**b**) elastic modulus and fracture dissipated energy of a PAM/GO single network (SN) gel, agar-PAM DN gel, and agar-PAM/GO DN gel. (**c**) Loading–unloading cyclic tensile stress–stain curves at a maximal strain of 1000%, and (**d**) corresponding dissipated energy of three gels. (**e**) Photos of the stretching process of agar-PAM/GO DN gel (from i to iii). Reproduced with permission from [48]. Copyright (2016) Advanced Engineering Materials.

Self-healing materials usually have poor mechanical properties, which limit their applications. In order to improve the mechanical behaviors, graphene and its derivates were usually added into the polymer matrix [36,52,63,67,81,100]. For instance, Deng and Wang et al. synthesized a gar-PAM/GO nanocomposite double network (DN) hydrogel with good fatigue resistance and self-healing ability [48]. The hydrogels exhibit excellent mechanical properties with a fracture strain of 4600%, fracture strength of 332 kPa and fracture dissipated energies of 11.5 MJ m^−3^. The healed hydrogels also have favorable mechanical properties with a fracture strain of 2000% and a fracture strength of 153 kPa. GO played a role as the cross-link agent in the polyacrylamide network, which improved the tensile property (Figure 11). 

The distribution of GO sheets in the composites affects the properties of the polymer composites especially the mechanical property. GO sheets were slightly agglomerated at high contents (0.5 wt %, 1.0 wt %). Primary GO sheets are well dispersed in the polymer matrix at low content (0.1 wt %). Sun et al. fabricated a covalent bonding GO/PU composite with significant mechanical reinforcement and thermal healable property. The Young’s modulus (21.95 ± 2.56 MPa) and fracture strain (449 ± 16%) increased twice and the fracture stress (8.01 ± 0.71 MPa) even increased nearly 4 times at a GO loading of only 0.1 wt %. The enhanced mechanical properties of the composites are ascribed to the good dispersion of GO and covalent linking based on DA chemistry [79]. To improve the mechanical properties, functional groups are usually introduced on the graphene surface. Liu et al. synthesized maleimide functionalized GO and then added it into a PU matrix, forming the self-healing composite [67]. The composites with maleimide functionalized GO exhibit higher self-healing efficiency and mechanical properties than the composites with unmodified GO because the maleimide functionalized GO serves as cross-linking points, improved the dispersion and interfacial interaction with the polymer matrix, which effectively dissipates energy and obviously increases the mechanical properties.

### 4.2. Shape Memory Property

**Figure 12:**
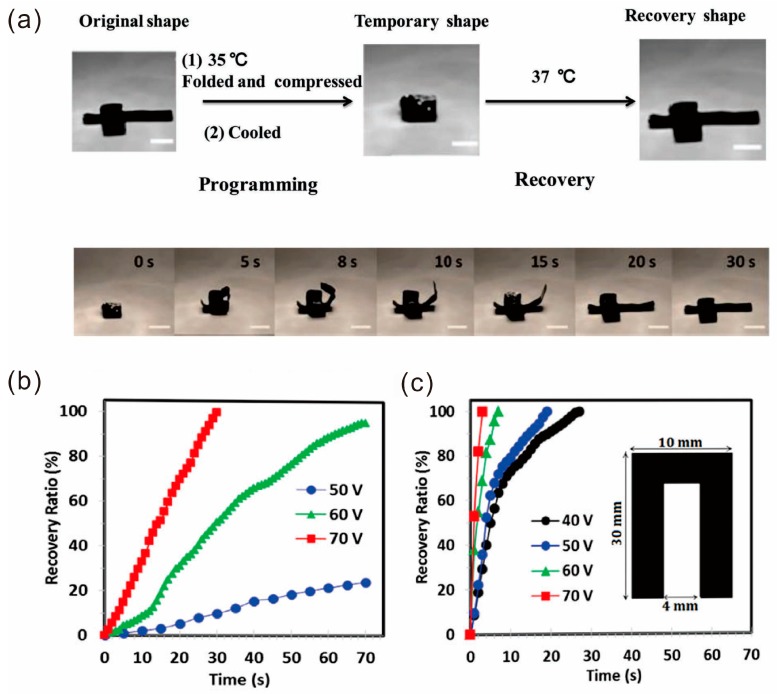
(**a**) The shape memory behavior of an unfolded cube box (10 mm × 10 mm × 10 mm) (top). Photographs demonstrating the shape memory behavior of the unfolded cube box in different times (bottom). Scale = 10 mm. Reproduced with permission from [80]. Copyright (2013) Macromolecular Rapid Communications. Electrically activated shape recovery ratio as a function of time under various triggering voltages for (**b**) PVAc/Gr3 and (**c**) PVAc/Gr4.5. The inset shows the sample geometry. Reproduced with permission from [41]. Copyright (2016) Society of Chemical Industry.

Besides high mechanical properties, shape memory behavior is an attractive property for self-healing graphene/polymer composites due to their wide applications in machinery, electronic device, chemical industry, biology and other fields [47,50,59,61,80,82]. For instance, Weng and Dai et al. prepared a series of graphene–poly(acrylamide-*co*-acrylic acid) hybrid materials with shape memory behavior and self-healing ability [80]. An unfolded cube box was designed as the original shape and heated at 35 °C. The compressed box can recover its original shape after heated at 37 °C for 30 s and can be repeated 10 times (Figure 12a). The hyperbranched PU–TiO_2_/RGO nanocomposite fabricated by Karak et al. also possessed excellent shape memory behavior [50]. As RGO is a conducting material, it absorbs the energy from sunlight and efficiently transfers the absorbed energy to the PU matrix. Therefore, the nanocomposite reaches its transition temperature easily to recover its shape. The surface temperature was measured to be 38.1–38.4 °C at the time of shape recovery. 

For conductive graphene/polymer composites, the shape memory can be realized by electrical conductivity. When the content of conductive filler in a polymer matrix exceeds the percolation threshold, the conductive network can generate Joule heating. Therefore, the speed of the shape memory depends on the electrical conductivity of the filler. For instance, Mohammad and coworkers synthesized fast electroactive shape memory and self-healing PVA/graphene nanocomposites. The electrically triggered shape recovery experiments of the composite containing 3 and 4.5 wt % of graphene (designated as PVA/Gr3 and PVA/Gr4.5, respectively) were conducted under four different DC voltages (40, 50, 60 and 70 V) and the resulting recovery ratio as a function of recovery time is presented in Figure 12b,c, respectively. It can be clearly seen that increasing the graphene content from 3 to 4.5 wt % leads to a dramatically faster recovery response [41].

### 4.3. Conductivity and Electrical Devices

Generally, a polymer is partially an insulator because the covalent bond of the polymer chain does not have free carries (electrons), on the other hand, as the polymer molecules pile together by van der Waals forces, the distances between the molecules are large, electrons overlap between the molecules are poor, so the free carries are very difficult to mobile in polymer [101]. Therefore, graphene can be used as an ideal filler to prepare conductive self-healing composite due to its inherent good conductivity [57,68,88,99,102]. The content of graphene highly affects the conductivity of self-healing composites and the conductivity of composites increases with the increase of graphene content. The charge-transfer resistance of a bPEI/(PAA-graphene) multilayer polyelectrolyte film electrode prepared by Ge et al. is 750 Ω, which is better than the pure bPEI/PAA electrode without graphene [76]. In conductive hydrogels, the water content of hydrogels is also a factor affecting the conductivity [103]. For example, Tang et al. fabricated a versatile hydrogel composite based on a commercial superabsorbent polymer and a hyperbranched polymer with RGO though a simple hydrothermal method [78]. The hydrogel possesses a super water-absorption ability and a fast electrical self-healing ability. The main factor influencing conductivity is the water content, and the RGO nanosheets improve the sensitivity of samples of water content because RGO can contribute to water dispersion and free ion (e.g., Na^+^) transportation.

**Figure 13:**
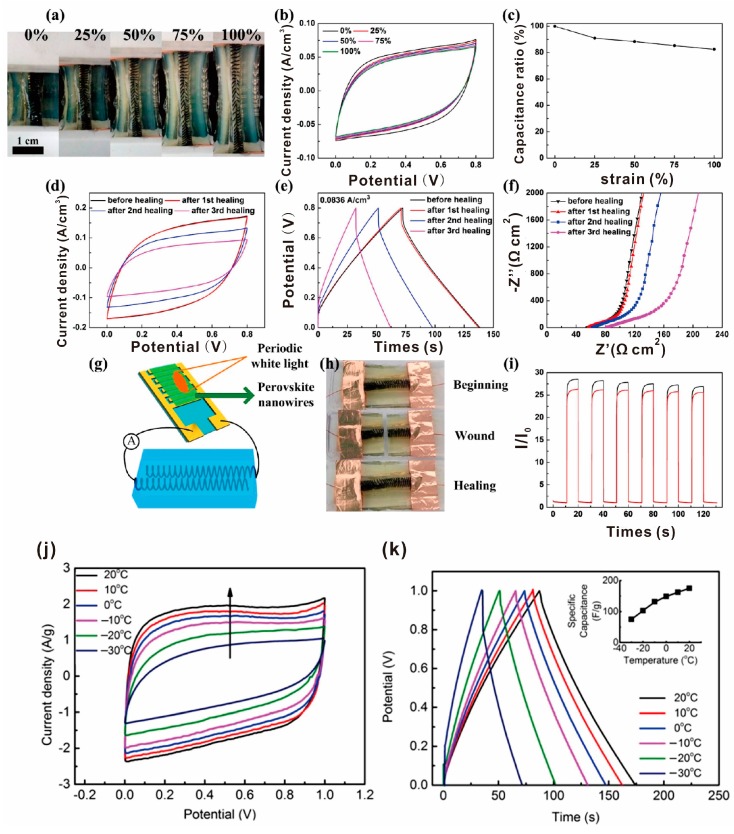
Electrochemical measurements and application for as-prepared stretchable and self-healing supercapacitors. (**a**) Photographs, (**b**) cyclic voltammogram curves, and (**c**) evolutions of specific capacitance of the supercapacitor before and after stretching to 100%. (**d**) Cyclic voltammogram curves, (**e**) galvonostatic charge−discharge measurements, and (**f**) Nyquist plots of the supercapacitor before healing and after self-healing cycles. (**g**) Illustration of the supercapacitor driving a photodetector of perovskite nanowires. (**h**) Photographs of the supercapacitor before and after self-healing. (**i**) Photocurrent dependence on time of the photodetector under illumination of on/off states driven by the original and self-healing supercapacitor after a healing cycle; red corresponds to the self-healing supercapacitor and black to the original. Reproduced with permission from [39]. Copyright (2017) ACS Nano. (**j**) Fabricated supercapacitor temperature dependence of cyclic voltammetry profiles at a scan rate of 20 mV s^−1^. The arrow indicates the direction of increasing temperatures. (**k**) Galvanostatic charging–discharging profiles at a current density of 1 A g^−1^. The inset indicates calculated specific capacitance with respect to temperature change. Reproduced with permission from [104]. Copyright (2017) Scientific Reports.

Supercapacitors are very common applications of conductive self-healing composite. For instance, Liu and Gao et al. assembled a stretchable and self-healable supercapacitor [39]. The supercapacitor was fabricated on two parallel RGO-based fiber springs wrapped with gel electrolyte and PU. After being cut and healed, the electrochemical performances of the device were still maintained at a high level (Figure 13a–i). Low temperature is a big challenge for applications of conductive self-healing composite. Chung and Ok et al. reported a supercapacitor with a high energy density that can work at low temperatures (even dropped to −30 °C) [104]. The supercapacitor was fabricated with a combination of biochar-RGO electrodes and a polyampholyte hydrogel. The reason why the supercapacitor performance is improved at low temperature is that water molecules strongly adsorbed on hydrophilic polymer chains cannot participate in ice formation (Figure 13j,k).

### 4.4. Anticorrosive Coating

**Figure 14:**
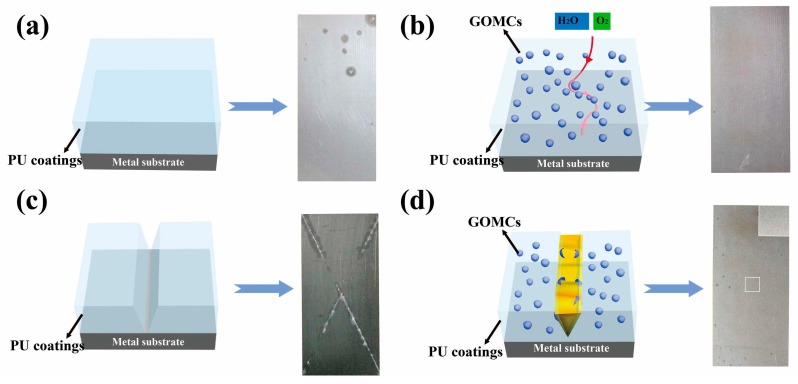
Schematic and images of (**a**) neat PU coating and (**b**) GOMCs/PU coatings subjected to the salt spray test for 116 h. Schematic and images of (**c**) neat PU coating and (**d**) GOMCs/PU coatings after scratching and 15 days of healing, subjected to the salt spray test for 43 h. Inset: the enlarged view of the white block. Reproduced with permission from [36]. Copyright (2016) Composites Science and Technology.

Fabrication of self-healing anticorrosive coatings has attracted attentions as it has the ability to extend the service life and prevent the substrate from corrosive attack. The self-healing coating on the surface of the material can repair the damaged surface automatically to protect the material [35,36,38,75,105,106,107,108,109,110]. For example, Lu and coworker reported a novel nanocomposite coating that consisted of lignin-modified graphene and waterborne PU [38]. The self-healing, electrically conductive coatings with UV resistant can be used as corrosion preventive or antistatic coatings. Gao et al. prepared a self-healing and anticorrosive coating [36]. The anticorrosion properties of neat PU coatings and GOMCs/PU composite coatings were characterized by the salt spray test on hot-dip galvanized steel (HDG) substrates with a 5% NaCl solution. The HDG plate coated with GOMCs/PU coatings showed no visual evidence of corrosion even after 116 h of salt spray test, while some white corrosion products were observed on the neat PU coated HDG plate (Figure 14).

### 4.5. Biological and Pharmaceutical Applications

One of the threats to human health is microbial contaminations or infections. Microbial fouling is the critical factor to degradation of polymeric self-healing materials. Therefore, antimicrobial activity is a fantastic property of self-healing composites. The nanocomposites prepared by Karak et al. exhibited good antimicrobial activity against Staphylococcus aureus, Escherichia coli and Candida albicans because sulfur-containing compounds and polysulfanes are generally considered to be antimicrobial agents [61].

**Figure 15:**
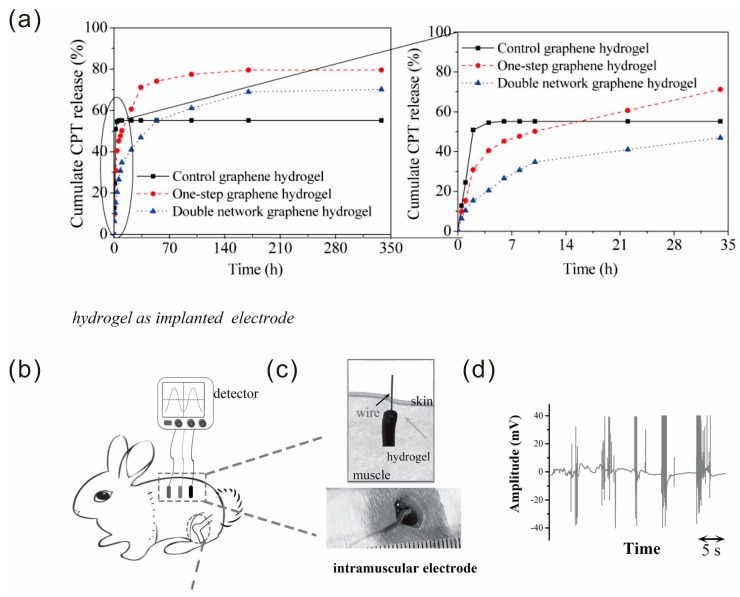
(**a**) Cumulate CPT release of hydrogels in PBS. Reproduced with permission from [53]. Copyright (2014) Materials Technology Advanced Performance Materials. The hydrogel as intramuscular electrodes. (**b**) Three hydrogel electrodes implanted into the dorsal muscle and the wires from the electrodes were transcutaneously connected to the signal detector. (**c**) Photos of the hydrogel implantation. (**d**) Example of the electromyographic signal recorded by the implanted hydrogel electrodes from the muscle when the rabbit was interfered by external stimulation. Reproduced with permission from [102]. Copyright (2016) Small.

The medical field also has great interest in self-healing materials. For example, Hu et al. fabricated a double network hydrogel based on β-CD functionalized graphene and *N*,*N*-dimethylacrylamide that can achieve a self-healing ability at 37 °C [53]. Camptothecin (CPT) as a model anticancer drug was loaded into the hydrogel before the second hydrogel network was introduced. The content of loaded CPT and the cumulated CPT release in a β-CD functionalized graphene hydrogel are both better than that of a pristine graphene hydrogel (Figure 15a). Therefore, this system had potential capacity as anticancer drug carrier. 

Due to the biocompatibility of DNA, the hydrogels can possess a variety of biological and environmental applications. Shi et al. reported GO/DNA self-healing hydrogels with high mechanical strength, excellent environmental stability, high dye-adsorption capacity and can be applied in tissue engineering, drug delivery, and removing organic pollutant [81]. 

Self-healing hydrogels were proposed to be used as biomaterials because of the capability of spontaneously healing any injury. Lu et al. prepared a mussel-inspired conductive, self-adhesive, and self-healing tough hydrogel [102]. The hydrogel could be implanted because of its good long-term biocompatibility and detection in vivo, which showed more accuracy in detecting/stimulating specific muscles in deep tissue. The intramuscular hydrogel electrodes yielded excellent signals from the dorsal muscle after implantation. The magnitude of the signals was in the range of 0.1–40 mV (Figure 15b–d), which was much higher than the signals detected by the surface electrodes.

## 5. Future Perspectives

Self-healing graphene/polymer composites are one of the most promising intelligent materials. The latest research progress in the preparation, properties and applications of self-healing graphene/polymer composites is reviewed and some of the composites are summarized in Table 1. Despite the rapid development of this research area, there are still many challenges that should be solved and considered in view of the practical applications.

(1) Graphene, as a component of the self-healing composites, can play an active role in the self-healing process because of the photothermal energy transformation ability. In this case, graphene acts as an energy absorber to transform irradiation or sunlight to thermal energy rapidly and efficiently to promote the mutual diffusion of the polymer chains across the damaged interface, thus facilitating the self-healing process. On the other hand, graphene in the composites is only used as a reinforcing agent to enhance the mechanical strength. In this case, the self-healing process may be impeded because some interactions between graphene and polymers will limit the movement and diffusion of the polymer chains. In addition, the glass transition temperature of the composites may be changed after the addition of graphene, thus influencing the self-healing efficiency. Therefore, graphene as a reinforcing agent can improve the mechanical performance. However, the self-healing efficiency of the composite will be reduced. The mechanical properties and self-healing efficiency are two contradictory aspects. How to balance the two aspects to achieve graphene/polymer composites with both high mechanical strength and high self-healing efficiency is a challenge.

(2) For intrinsically self-healing graphene-based materials, simple contacting without any external stimuli is considered to be most potential considering practical application. Therefore, exploring this kind of self-healing graphene-based composites is desired.

(3) For electrical conductive graphene/polymer composites, the mechanical strength and the conductivity are two contradictory aspects. Increasing the graphene content will increase the conductivity of the composite, but excess graphene will reduce the mechanical strength. How to obtain high mechanical strength and high conductivity of the graphene/composites with self-healing properties is a challenge.

(4) A lot of polymeric materials should be developed which exhibit self-healing properties. Also, it is highly challenging to endow the combined properties in a single material. The structural incompatibility among such types of polymers is one of the crucial reasons for this difficulty.

(5) To improve the compatibility of polymer and graphene, graphene usually needs to be modified. However, modification of graphene will lose some inherent properties of graphene. Therefore, how to improve the compatibility of polymer and graphene and retain the inherent properties of graphene as much as possible in the composite is a challenge.

## 6. Conclusions

In this review, we have summarized the recent progress of graphene-containing polymer composites with self-healing capability. The preparation methods, self-healing conditions, and properties and applications of the graphene/polymer composites have been briefly discussed. Finally, the further perspectives of the composites have been proposed. Intelligent materials and self-healing materials, specifically for graphene-containing composites, are still in the initial stage. There will be a great research space in the field. The progress will guide further development of the self-healing graphene/polymer composites.

## Figures and Tables

**Table 1:** The properties of various self-healing graphene/polymers composites.

Materials	Self-Healing Mechanisms	Self-Healing Condition	Self-Healing Efficiency	Original Mechanical Property	Applications	Reference
FG/TPU material	PU chains diffuse	IR light, Electricity, Microwave (2.45 GHz)	98% of electrical conductivity	Tensile strength 40 MPa	Transport industries, construction industries, electronics	[91]
P(AM-*co*-DAC)/GO Hydrogels	Hydrogen bonds, electrostatic interaction	Drop water	>92% of tensile strength, >99% of tensile strain and >93% of toughness	Young’s modulus 1 MPa Tensile strength 2 MPa	-	[46]
Chitosan/GO Hydrogel	π-π stacking, hydrogen bonds	Contact (room temperature)	-	Adhesive strength 1 MPa compressive stress 14 KPa	Electroactive tissue engineering applications	[95]
PU-DA-mGO	Dynamic covalent bonds	Heating (120 °C after 10 min)	90% of tensile strength	Stress 38 MPa	Aerospace, automobile, coating, electronics, energy, etc.	[67]
Graphene/PU	Diels-Alder chemistry	IR	96% of tensile strength	Breaking strength 36 MPa Young’s modulus 127 MPa	Flexible electronics	[68]
PVAc/graphene nanocomposites	Diffusion of the polymer chains	60 °C for 1 h	89% (mechanical properties)	-	Sensors and fast deployable and actuating devices	[41]
RFGO/PU composites	Diels–Alder chemistry	Microwaves	93% (mechanical properties)	Stress 24 MPa Young’s module 52 MPa	Flexible conductors, strain sensors	[63]
PAA-GO-Fe^3+^ Hydrogel	Ionic binding	Contact and immersed in FeCl_3_/HCl	Nearly 100% tensile	Tensile strength 2.5 MPa Elongation 700%	Soft actuators, robots	[47]
PDA-pGO-PAM hydrogel	Non-covalent bonds	Contact	60% of tensile strength 95% of electrical conductivity	Tensile strength 75 kPa	Bioelectronics	[102]
PAM/GO DN gel	Hydrogen bond	Heating 80 °C for 3 h	48% of tensile strength	Elongation 4600% Fracture strength 332 kPa	Engineering fields	[48]
Au@PCLx/rGO/Ag	Fibers soften and flow	Light irradiation (532 nm)	90% of tensile strength 91% of conductivity	Tensile strength 4.85 MPa	Optoelectronic devices	[88]
SR/GNP composite	Reversible bonds	Thermal annealing	87% of tensile strength	Stress 1.3 MPa	Seals, hoses and automotive sector	[43]
HPU-IO-RGO	Diffusion of the polymer chains	Microwave sunlight	99%	Tensile strength 24.15 MPa Tensile modulus 28.55 MPa Toughness 110.8 MJ m^3^	Transport, construction, electronics	[59]
GO-Clay-PDMAA Hybrid Hydrogels	Diffusion of the polymer chains hydrogen bonds	NIR	96% (mechanical strength)	Strength 184 kPa Elongation 1890%	Surgical dressing	[52]
GO/PU composites	Covalent bonding	Heating	78% of tensile stress	Tensile modulus 21.95 MPa Fracture stress 8 MPa Young’s modulus 22 MPa	Smart materials and structural material	[79]
SHPU/grapheme composites	Interchain diffusion	NIR	39%	Stress 4 MPa	Functional polymer	[62]
GO/PAA composite hydrogels	Diffusion of polymer chains hydrogen bond	Contact at different temperatures	88% (mechanical properties)	Tensile strength 0.35 MPa Elongation 4900% (healed)	Biomedical and engineering fields	[56]
